# 3D‐Printed Magnetoelectronics for Interactive Appliances and Self‐Aware 4D‐Printed Mechatronics

**DOI:** 10.1002/advs.75985

**Published:** 2026-06-13

**Authors:** Eduardo Sergio Oliveros‐Mata, Anna Martin Vilardell, Fabian Ganss, Christoph Leyens, Lukas Stepien, Denys Makarov

**Affiliations:** ^1^ Institute of Ion Beam Physics and Materials Research Helmholtz‐Zentrum Dresden‐Rossendorf e.V. Dresden Germany; ^2^ Fraunhofer Institute for Material and Beam Technology IWS Dresden Germany; ^3^ Institute of Materials Science Technische Universität Dresden Dresden Germany

**Keywords:** 3D printed magnetic field sensors, 3D printing, 4D‐printed mechatronics

## Abstract

Additive manufacturing enables fabrication of intricate electronic devices embedded in complex‐shaped structural components. Here, we add a new member to the family of 3D‐printed electronics ‐ a high‐performance 3D‐printed magnetic field sensor featuring more than 300% magnetoimpedance effect at low frequencies and single point magnetic vector field reconstruction relying on 3D Hall effect magnetometry. The sensors are shaped as mechanically flexible and magnetically controllable springs as well as 3D crosses demonstrating abilities in tailoring the sensor response, operation field range, and operation frequency through rational design of the sensor geometry. The application potential of 3D‐printed magnetoelectronics is featured via magnetic toggle switches for smart home, continuous joysticks for robotics control, three‐axis magnetometers for volumetric multi‐point detection, and self‐aware 4D‐printed mechatronic actuators. This technology enables 4D‐printed structures that fold motion sensing directly into their design and enable each part to interact intelligently within a larger mechanism being aware of their environment and user interactions.

## Graphical abstract

3D‐printed magnetoelectronics integrate high‐performance magnetic field sensing directly into complex structural components. Flexible spring and cross‐shaped sensors exhibit giant magnetoimpedance and 3D Hall sensing for vector field reconstruction. Applications include smart‐home switches, robotic joysticks, volumetric magnetometers, and self‐aware 4D‐printed mechatronics with embedded motion and interaction sensing.

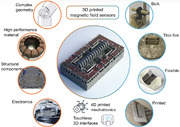

## 1 Introduction

Additive manufacturing, commonly known as 3D printing, has experienced remarkable growth, transitioning beyond the fabrication of structural components toward increasingly complex functional and electronic devices [1, 2, 3, 4]. Relying on 3D printing technology, sensing capabilities can be directly integrated into manufactured parts, achieving higher functionality, automation, and reduced post‐assembly processing [5]. The family of additively manufactured sensors focuses primarily on the assessment of mechanical properties with directional sensitivity and selectivity, including force, pressure, and torque [6, 7, 8]. However, 3D‐printed structures still largely lack integrated magnetic field sensing capabilities.

Magnetic sensing offers distinct technological advantages for 3D‐printed electronic, mechatronic, and 4D‐printed systems. It enables contactless detection of proximity, rotation, and position, interaction through non‐magnetic barriers, and intrinsic feedback for actuating structures without requiring mechanical contact or external add‐on sensors [9, 10, 11]. When 3D‐printed structures are designed to respond to external stimuli over time, they are referred to as 4D‐printed systems, where the “fourth dimension” represents the temporal change in shape or functionality [12]. These dynamically changing complex‐shaped objects require embedded motion sensors for their real time control and monitoring. For conventional actuators, these tasks are usually addressed using magnetic field sensors [11, 12, 13]. At present, 3D‐printed structures do not possess magnetic sensitivity.

**FIGURE 1:**
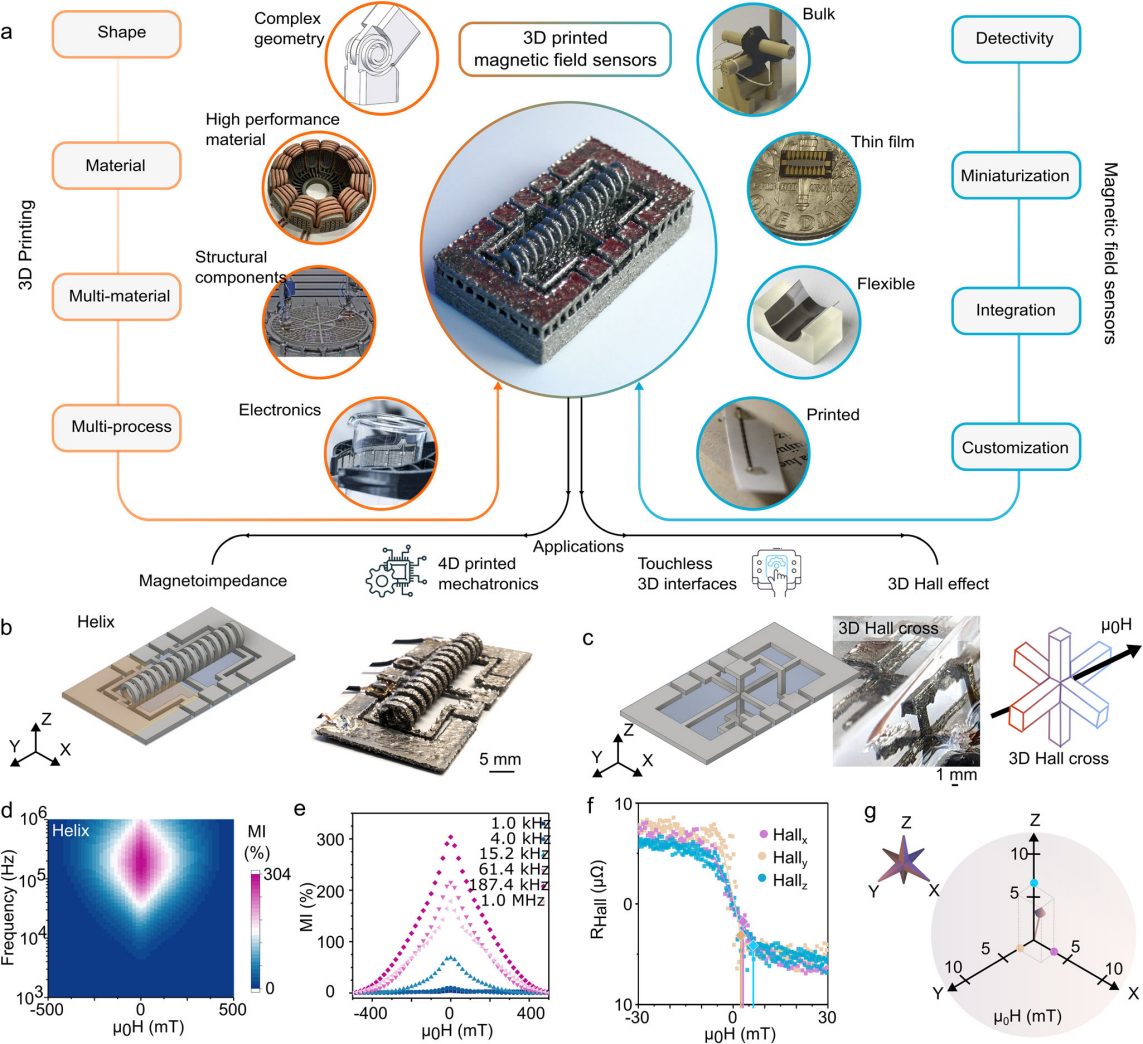
(a) Convergence of additive manufacturing and magnetoelectronics. (b) Design geometry and 3D‐printed realization of a helix‐shaped structure for 4D actuation and magnetic field sensing. The structure has 15 turns, outer diameter *D* = 5 mm, inner diameter *d* = 3 mm, and length *l* = 25 mm. For sensing, the 3D‐printed sensor structure has six 3D‐printed electrical contacts for magnetoimpedance and Hall effect measurements. (c) 3D‐printed 3D Hall cross consisting of three mutually orthogonal conductive segments. (d) Frequency‐ and magnetic field‐dependent magnetoimpedance ratio with the maximum value of 304% at 187 kHz measured from a 3D‐printed sensor shown in panel (b). (e) Representative magnetoimpedance curves taken from the map shown in panel (d) for several selected driving frequencies. (f) Measurement of the magnetic field along three sensing axes of the 3D‐printed 3D Hall cross shown in panel (c). (g) A vector representation of the magnetic field applied as $\mu_{0}H=\left(3.1,\;2.4,\;6.4\right)\;\mathrm{mT}$ reconstructed using the 3D‐printed 3D Hall sensor. The image credits in panel (a) are in the counterclockwise order: Reproduced from Lussenburg [20], CC BY 4.0; © Fraunhofer IGCV, all rights reserved; © Fraunhofer IWS, all rights reserved; © Fraunhofer IWS, all rights reserved; reproduced from Oliveros [10], CC BY 4.0; reproduced from Makushko [17], CC BY 4.0; reproduced from Nhalil [16], CC BY 4.0; reproduced from Coillot [15], CC BY 3.0.

Achieving magnetic field sensing functionalities directly in 3D‐printed structures is a new and yet to be explored topic in unconventional magnetoelectronics. Modern magnetoelectronics has evolved from bulky devices through rigid thin‐film sensors to mechanically flexible and printed sensing elements [10, 14, 15, 16, 17]. In particular, flexible and printed magnetoelectronics enabled applications in emerging areas of soft robotics, human‐machine interfaces, and electromobility [14, 18, 19]. Figure 1a illustrates the concept of monolithic 3D‐printed magnetic field sensors as a route toward magnetically responsive 3D‐printed mechatronics and touchless 3D interfaces. Such convergence of magnetically aware 3D‐printed systems could enable continuously variable custom joysticks, motors with built‐in speed sensing, robotic arms with intrinsic actuation awareness, and self‐aware 4D‐printed actuator structures. In this work, we use the term *self‐aware* to describe a mechatronic structure that can directly sense its own actuation state, such as position or deformation, via its intrinsic electrical signal response, without requiring external add‐on sensors; this sensing information can subsequently be used for monitoring and closed‐loop control.

An integration of magnetic field sensors directly into 3D‐printed electronic parts is challenging, as the majority of high performance magnetic field sensors are based on thin films, including various types of magnetoresistive and Hall effect sensors [21]. Notably, specialized nanoscale processing such as focused electron beam induced deposition (FEBID) has enabled 3D‐shaped magnetic field sensors at the nanometer scale [9, 22, 23, 24, 25]. However, these physical effects, which function well at the nanoscale, often diminish or become impractical in mm‐size samples, which are typical for conventional 3D‐printed parts. While there are examples of volumetric sensors, they are often based on 2D sensing structures with transducing readout mechanisms that require additional elements hardly compatible with 3D printing processes [26, 27, 28, 29, 30]. Therefore, the realization of 3D‐printed monolithic magnetosensitive structures is still pending.

Here, we realize monolithic 3D‐printed magnetic field sensors and demonstrate their direct integration into 3D‐printed electronic and mechatronic parts. These sensors reveal magnetoimpedance and Hall effects and enable geometry‐tailored magnetic field detection in complex three‐dimensional structures. The laser powder bed fusion (L‐PBF) was applied to 3D‐print magnetic ${\text{Fe}}_{64}{\text{Ni}}_{36}$ alloy powders into complex shaped structures resembling mechanically flexible springs and 3D crosses, successfully demonstrating the ability to tailor the sensor response and functionalities through geometry. We report the optimal laser parameters to obtain dense and mechanically robust parts with sub‐mm resolution. Finally, we present different proof‐of‐concept applications, such as movable switches for smart home, custom continuous joysticks for robotics control, three‐axis magnetometer interfaces for volumetric multi‐point detection, and self‐aware 4D‐printed mechatronic actuator systems for optomechanical parts.

## 2 Results and Discussions

A convergence of printability, conductivity, and sensitivity in additively manufactured materials and particularly alloys is known to be a challenge [31, 32, 33]. At the same time, the best materials for high‐performance magnetic field sensors are CoFe‐ and FeNi‐based alloys [34, 35, 36]. For this work we chose ${\text{Fe}}_{64}{\text{Ni}}_{36}$ alloys due to their low thermal expansion coefficient, high magnetic permeability, and metallic conductivity. While there are studies characterizing the mechanical, microstructural and corrosion properties of 3D‐printed ${\text{Fe}}_{64}{\text{Ni}}_{36}$ alloys [37, 38, 39, 40, 41, 42], their potential for magnetic field sensing and 4D printing has not been explored yet.

### 2.1 3D Printing of Magnetic Field Sensors

We applied L‐PBF of ${\text{Fe}}_{64}{\text{Ni}}_{36}$ alloy powders to 3D‐print parts of complex shapes with sub‐mm features and recurring overhangs, such as helical coiled structures for 4D actuation (Figure 1b). The magnetoimpedance of helix‐shaped actuator structures with 15 turns, outer diameter *D* = 5 mm, inner diameter *d* = 3 mm, and length *l* = 25 mm, achieved a peak 304% change of the electrical impedance at 187 kHz and 80 mA driving current (Figure 1d). The magnetoimpedance curves at different frequencies demonstrate a single peaked profile that facilitates the use of these devices as a sensor of the magnetic field strength in a broad range of frequencies (Figure 1e).

Furthermore, we designed 3D‐printed 3D Hall crosses (Figure 1c) consisting of three mutually orthogonal conductive segments. The three‐dimensional design capabilities allow us to simplify the construction of a structure with orthogonal current flow directions. While 2D lithography and 2D printing methods can achieve single plane current direction control, our 3D‐printed structure enables seamless switching of the current direction in three orthogonal planes allowing to access the three‐axis magnetic field components by monitoring the respective Hall responses. The data shown in Figure 1f provide access to the sensitivity $S=\left(0.75,\;2.68,\;0.86\right)\;m\Omega \;T^{-1}$ and detectivity $d=\left(400,\;111,\;348\right)\;\mu T\;{\mathrm{Hz}}^{-1/2}$ of the Hall effect sensor along three orthogonal directions. We note that the sensitivity components were extracted from the linear slopes of the Hall response, $S_{i}=dR_{H}/d\left(\mu_{0}H_{i}\right)$ with i=x,y,z, obtained by linear interpolation in the low‐field range of ±5mT. The corresponding detectivity components were calculated as $d_{i}=R_{\mathrm{noise}}\left(1\;\mathrm{Hz}\right)/S_{i}$, where $R_{\mathrm{noise}}\left(1\;\mathrm{Hz}\right)$ is the averaged resistance noise spectral density evaluated at 1 Hz for 30 s without field. The enhanced sensitivity along the y‐axis is attributed to anisotropic magnetic‐field focusing introduced by the anisotropic contact layout. Notice that a larger mass of printed material is aligned across the y‐axis, which preferentially increases the effective magnetic field component sensed along this direction. The sensor is able to track independently the three magnetic field components and deliver a measurement of the strength and direction of an arbitrary applied magnetic field. A Cartesian representation in Figure 1e illustrates an example measurement of the magnetic field components when the field is applied as $\mu_{0}H=\left(3.1,\;2.4,\;6.4\right)\;\mathrm{mT}$, with magnitude $|\mu_{0}H|=7.5\;\mathrm{mT}$ and unit‐vector direction $\hat{H}=\left(0.413,\;0.320,\;0.853\right)$ showcasing the capability of vector measurements of magnetic fields using a 3D‐printed 3D Hall effect sensor.

### 2.2 Screening the L‐PBF Printing Parameters

**FIGURE 2:**
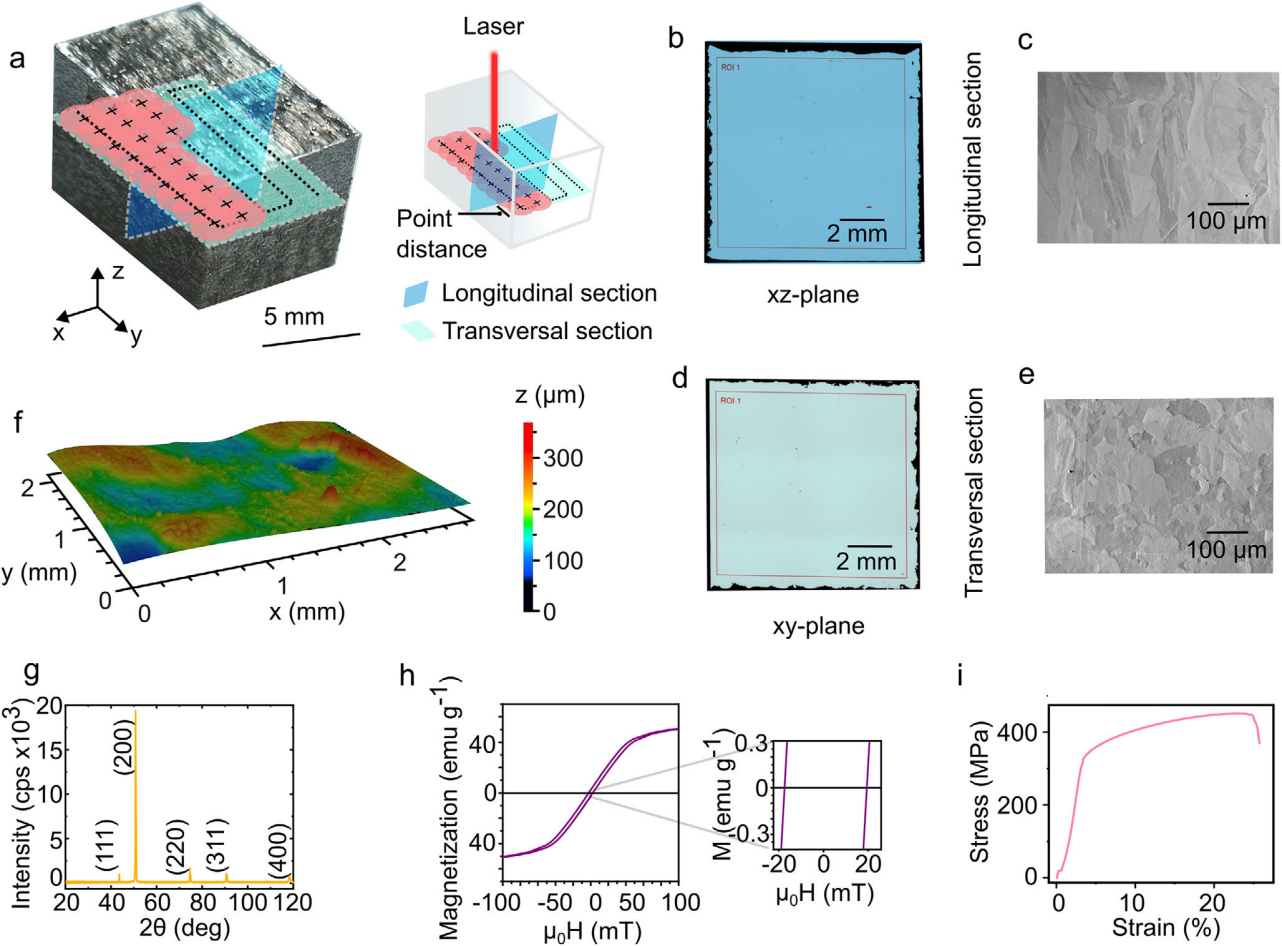
Screening the L‐PBF printing parameters and characterization of 3D‐printed ${\text{Fe}}_{64}{\text{Ni}}_{36}$ alloy parts. (a) A representative 3D‐printed ${\text{Fe}}_{64}{\text{Ni}}_{36}$ alloy cube produced through L‐PBF. Near‐fully dense structure in (b) longitudinal and (d) transversal cross‐sections of the printed cube. (c) Longitudinal and (e) transversal cross‐section micrographs showing columnar grain structure aligned along the building direction (z‐axis). (f) Topographic map of the as‐printed surface with the surface roughness $S_{a}$ = (32±1) μm. (g) XRD pattern of the 3D‐printed cube (xy‐face) confirming the fcc γ‐phase structure. The XRD pattern of the powder material is provided in Figure S2c. (h) Magnetic hysteresis loop taken at room temperature. (i) Stress‐strain curve showing ultimate tensile strength of (402±6) MPa and elongation at break of (30±3)%.

Due to rapid temperature changes during fabrication, additive manufacturing of metallic alloys frequently results in materials with high defect densities, limiting their 3D‐printability to only a few out of the approximately 5500 known alloys [32, 43]. Figure 2 shows the fabrication and characterization of dense, high‐quality ${\text{Fe}}_{64}{\text{Ni}}_{36}$ alloy parts fabricated using L‐PBF that allowed us to obtain sub‐mm feature sizes. A summary of the characterization of the ${\text{Fe}}_{64}{\text{Ni}}_{36}$ alloy powder is given in Figures S1 and S2. A representative ${\text{Fe}}_{64}{\text{Ni}}_{36}$ alloy cube produced through this additive manufacturing process is shown in Figure 2a. To obtain high density structures, a factorial design of experiments was carried out by screening the laser power, exposure time, and hatch distance (see Methods for details). The optimal process parameters were considered the ones that resulted in the highest density of 99.95% of the 3D‐printed alloy, obtained with the exposure time of 100 μs and a hatch distance of 110 μm, corresponding to a volumetric energy density of 94.7 J ${\mathrm{mm}}^{-3}$. The optimization process described herein was primarily focused on the laser powder bed fusion parameters to ensure the fabrication of near‐fully dense and structurally sound ${\text{Fe}}_{64}{\text{Ni}}_{36}$ components. Following this structural optimization, representative sensor geometries were fabricated and characterized for their magnetic sensing performance. The reported magnetoimpedance and Hall effect data reflect the performance of these devices fabricated under the established optimal printing conditions. Figure 2b,d reveal a near‐fully dense structure in both longitudinal and transversal cross section views of the cube where porosity fluctuates between 2.02% and 0.38%, respectively. A summary of printing parameters and SEM micrographs showing line profile structures is provided in Table S1.

Figure 2c and Figure S3 show longitudinal cross‐section micrographs with a distinct columnar grain structure aligned along the building direction (z‐axis). This preferential grain orientation is a result of the directional heat flow inherent to the L‐PBF process where grains cross several deposited layers and have a width of less than 100 μm. On the other hand, the transversal cross‐section reveals a more isotropic grain structure. This observation suggests that while the grain growth is strongly influenced by the printing direction, the microstructure remains rather uniform in the plane perpendicular to the building direction. Figure 2f presents a topographic map of the as‐printed surface revealing the surface roughness, $S_{a}$, of (32±1) μm, typical for L‐PBF‐fabricated components. The XRD pattern confirms a predominance of the face‐centered cubic (fcc) γ‐phase structure of the ${\text{Fe}}_{64}{\text{Ni}}_{36}$ alloy (Figure 2g) without detectable secondary phases, indicating that the desired crystallographic structure of the alloy was retained upon L‐PBF printing process.

Figures 2h,i summarize the functional properties of the printed structures. The magnetic hysteresis loop taken at room temperature reveals the coercive field of $H_{c}=2.3$ mT, saturation magnetization $M_{s}=116.7$ emu $g^{-1}$, and relative susceptibility $\chi_{r}=333$ (Figure 2h). The stress‐strain curve of a 3D‐printed specimen fabricated under optimal printing conditions provides insights into the mechanical behavior of the material, showing the ultimate tensile strength of (402±6) MPa and the elongation at break of (30±3)% (Figure 2i). Additional hardness measurements of (167±6) HV0.3 were taken of the part printed with the optimal conditions. These values are typical for ${\text{Fe}}_{64}{\text{Ni}}_{36}$ alloys and highlight their potential as a soft magnetic material not only for magnetic field sensing but also for mechanical components [44].

### 2.3 Magnetoimpedance Performance

**FIGURE 3:**
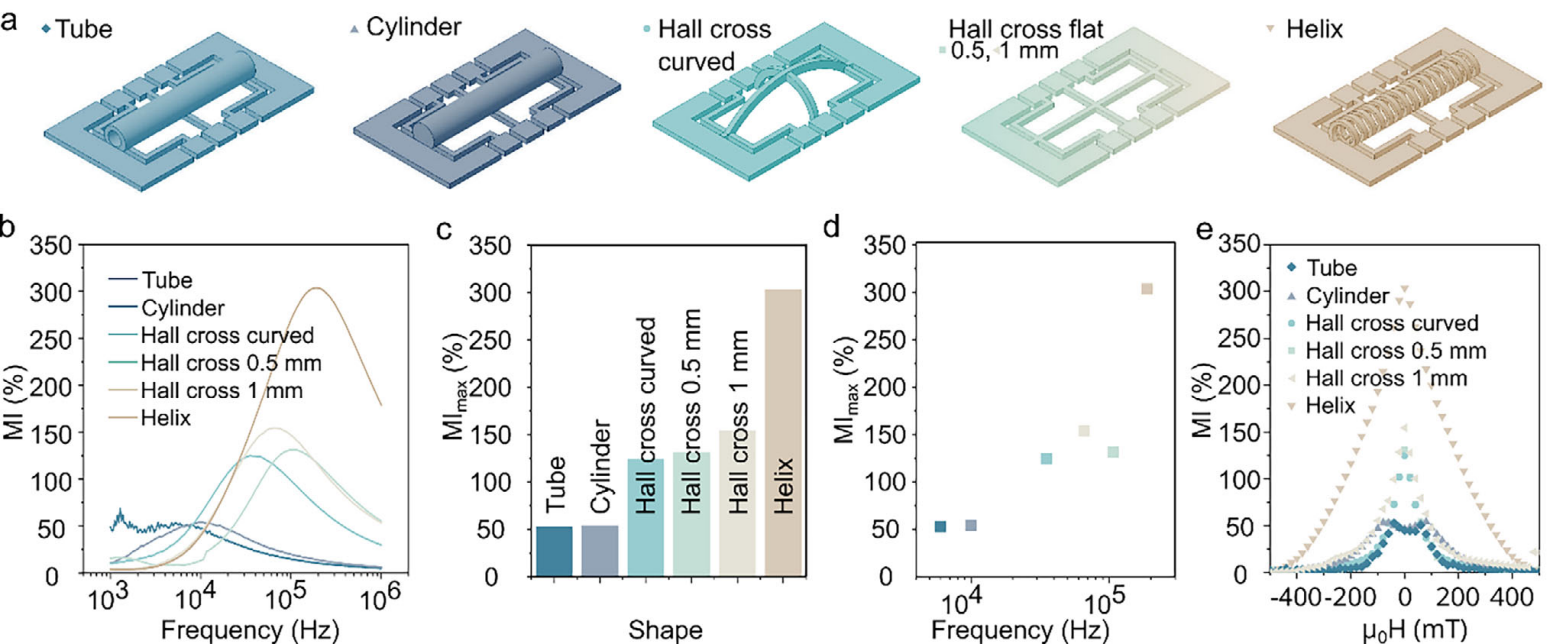
Magnetoimpedance response of 3D‐printed ${\text{Fe}}_{64}{\text{Ni}}_{36}$ sensors. (a) Schematics of different geometries: tube, cylinder, curved segment, straight segment, and helix. (b) Magnetoimpedance response peaks at frequencies in the 10–200 kHz range. The frequency corresponding to the maximum effect is dependent on the 3D‐printed geometry. (c,d) Maximum magnetoimpedance values and corresponding frequencies for each sensor geometry. (e) Representative magnetoimpedance profiles showing maximum amplitude change as a function of the applied magnetic field. Helix geometry shows the highest maximum magnetoimpedance ratio and largest dynamic range.

Figure 3 represents the influence of the sensor geometry on the magnetic field sensing response of 3D‐printed ${\text{Fe}}_{64}{\text{Ni}}_{36}$ structures. Figure 3a and Figure S4 display schematics of the different geometries that have been studied, which include tube, cylinder, helices, curved and straight segments of different thickness (0.5 and 1 mm thickness). The magnetoimpedance spectra for these structures measured over a frequency range of 1 kHz‐1 MHz and a magnetic field range of ±500 mT are shown in Figure S5. The magnetoimpedance ratio peaks at frequencies in the 10–200 kHz range with the precise frequency corresponding to the maximum of the effect being dependent on the sensor geometry (Figure 3b). Figure 3c,d summarizes the extracted maximum magnetoimpedance values for each geometry and their corresponding frequencies, respectively. We observed a quasi‐linear relationship between the maximum magnetoimpedance ratio and the frequency at which it occurs for different geometries. Figure 3e displays representative magnetoimpedance profiles when the maximum amplitude change is achieved as a function of the applied magnetic field. We observed that, among the studied geometries, the samples with the largest magnetoimpedance effect tend to show their maximum response at higher frequencies. In a basic description of magnetoimpedance, the characteristic frequency is commonly related to the skin‐depth condition and therefore to the conductor cross‐section and magnetic permeability. However, our results suggest that these parameters alone do not fully describe the response of the present 3D‐printed geometries. For example, elements with nominally identical cross‐section, such as the helix and the 1 mm Hall cross, show different values of ${\mathrm{MI}}_{\max}$ and $f_{\max}$. This suggests that the topology of the conductive path also influences the MI response. Further experiments are needed to separate these contributions systematically. In particular, control geometries with identical conductive path length and cross‐section, but without helical winding, such as meanders or long straight paths, as well as series with different thicknesses, would help to determine whether this trend holds and which specific geometric parameters control or violate this correlation. This would be relevant for the design and tunability of both the MI magnitude and the optimum operating frequency. From the geometries under evaluation, the helix geometry shows the highest maximum magnetoimpedance ratio and the largest dynamic range. The helical (spring‐type) geometry was chosen as it co‐optimizes mechanical compliance and electromagnetic coupling. Mechanically, the helix acts as a tunable elastic element enabling measurable, reversible out‐of‐plane displacements under magnetic forces. Electromagnetically, the helical topology creates a coil‐like current path that increases the effective interaction length between the current and the applied magnetic field, enhancing the magnetoimpedance response. This behavior originates from the interplay between the AC current path and the local magnetic anisotropy induced by the 3D geometry. Due to its inherent coil‐like topology, the helical current path is predominantly perpendicular to the longitudinal magnetic field, maximizing the interaction with the transversal magnetization components. In contrast, straight wires maintain a parallel configuration between the current and the axial field, leading to a sharp, high‐sensitivity peak at low fields [34]. Notably, tube and cylinder geometries with smaller aspect ratios exhibit double‐peak profiles, which are characteristic of a transversal easy‐axis anisotropy where the magnetization must be rotated out of its preferred orientation by the external field before the maximum impedance change occurs [34, 45]. On the other hand, the always parallel current and magnetization directions in the straight wires result in a sharp, well‐defined peak, reflecting high sensitivity and precision in the detection of small magnetic fields. Notably, a smaller aspect‐ratio tube and cylinder geometries exhibit moderate magnetoimpedance performance with characteristic double‐peak profiles, typical of transversal easy‐axis anisotropy.

Notably, the maximum magnetoimpedance ratio of the 3D‐printed sensor structures investigated in this work corresponds to frequencies considerably lower than those reported for sensors based on thin films, ribbons, or wires (typically above 1 MHz  [34, 45, 46, 47]). Historically, optimized amorphous and composite wires reached MI ratios on the order of 1200% in the MHz range [48]. Recent advances in engineered structures have pushed these values significantly further; for instance, a spiral‐structured Co‐based microwire recently achieved a record MI ratio of $∼4.3\times 10^{4}$% by exploiting combined material and circuit resonance effects [49]. Specialized composite and trilayer configurations, such as magnetic/conductor multilayers, have attained extraordinarily high MI at lower frequencies–e.g., ∼830% at ∼0.3 MHz and ∼600% at ∼0.1 MHz–though these typically require carefully tuned geometries, multilayer stacking, and specific post‐processing like field annealing [50]. State‐of‐the‐art multilayer thin films consisting of ferromagnetic layers interleaved with insulators and conductors can reach MI ratios of ∼700% at tens of MHz [51]. In contrast, more integrated Permalloy thin‐film sensors on rigid substrates report MI ratios around 300%–350% under optimized conditions. In this context, our 3D‐printed ${\text{Fe}}_{64}{\text{Ni}}_{36}$ sensors demonstrate MI ratios >300% at low frequencies (<200 kHz), which is comparable to high‐performance crystalline bulk alloys and exceeds the typical performance of standard amorphous ribbons in the same low‐frequency range. This lower frequency operation of 3D‐printed magnetoimpedance sensors simplifies the design of conditioning circuitry and reduces the implementation efforts due to the possibility of using cost‐efficient low‐frequency electronics.

A possibility for the geometric tunability of the magnetoimpedance responses offers appealing opportunities in terms of the tailoring sensing parameters like operating frequency, dynamic range, and maximum sensitivity range. For example, detecting a larger range of magnetic fields hold particular promise in applications such as robotics, where a strong magnetic field generated to move an electromagnetic actuator needs to be tracked precisely during the full magnetic operation range. The capability for modifying these properties at the design stage makes these 3D‐printed sensors highly adaptable to specific application requirements.

### 2.4 Three‐Axis Magnetic Field Tracking Using a Monolithic 3D‐Printed Hall Cross Structure

**FIGURE 4:**
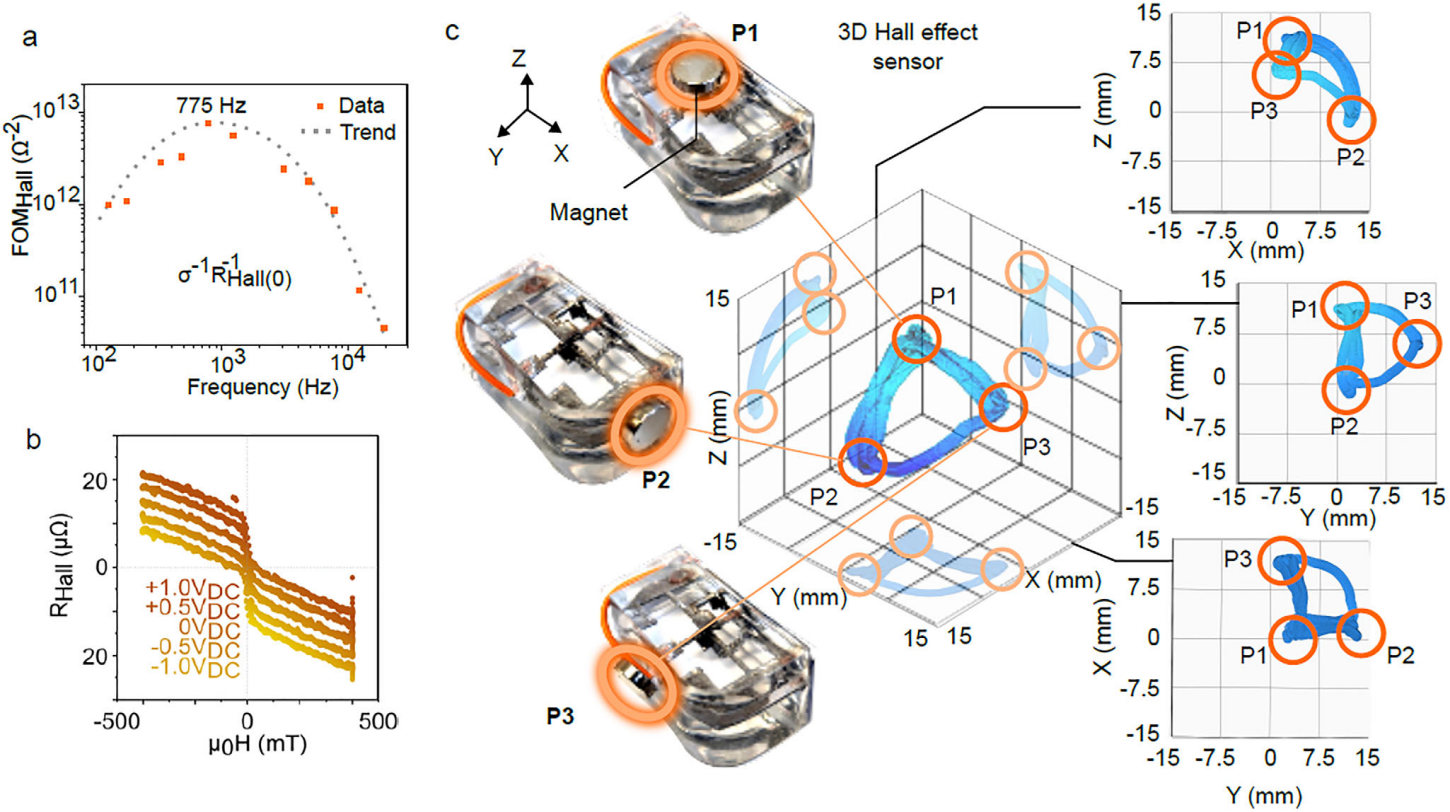
3D‐printed 3D Hall effect sensors. (a) Tailoring the figure of merit (FOM) of the Hall sensor showing the best performance at 775 Hz. The FOM is calculated as the inverse of the product of the sensor offset and the standard deviation of the Hall signal accumulated over a 30 s measurement interval. (b) Fine‐tuning of the Hall signal offset with an additional DC voltage bias. (c) 3D tracking demonstration showing a reconstruction of the position of a permanent magnet moved between three defined locations P1, P2, P3 following a 3D trajectory (blue trace in the central part of the panel) and the corresponding 2D projections onto XZ, YZ, XY planes (right part of the panel).

Beyond the field strength measurements based on the magnetoimpedance effect, 3D‐printed ${\text{Fe}}_{64}{\text{Ni}}_{36}$ sensors can also detect the full magnetic field vector by making use of the Hall effect. The intrinsic freedom of the design allowed by 3D printing using L‐PBF enables our sensing devices to independently measure all three components of the magnetic field vector in one single monolithic structure. As a proof of concept, we fabricated a three‐dimensional Hall cross structure that allows current to flow through different branches of the cross to selectively sense the three orthogonal magnetic field components (Figure 4). To maximize the performance of the sensor, we determined the operation frequency at which the sensor minimizes the offset in the Hall resistance and possess a lower noise (Figure S6). These factors were combined into one performance parameter, the figure of merit (FOM), which was calculated by taking the inverse of the product of the offset and the standard deviation of the Hall signal accumulated over a 30 s acquisition interval (Figure 4a). The higher the FOM metric is, the higher the stability and capability of detection of the sensor. The sensor showed the best performance at 775 Hz, which was used for the experiments discussed in the following. Figure 4b shows that by adding an additional DC voltage to the AC driving signal, further fine‐tuning of the Hall signal offset can be done, improving the sensor performance. The shape profile of the curve in Figure 4b corresponds to contributions from both the ordinary Hall effect (dominant at high fields after magnetic saturation) and the anomalous Hall effect (dominant at low fields via the sample magnetization), as reflected by the different slopes. In the present geometry and measurement configuration, planar Hall contributions are not expected to dominate the signal.

Figure 4c presents the use of a 3D‐printed sensor of the vector magnetic field for applications of multi‐axis spatial tracking of a magnetic field source (Video S1). This figure panel depicts the ability of the sensor to reconstruct the position of a permanent magnet moved in 3D space among three defined positions P1, P2, and P3. In the 3D plot in the center of the panel, the intersection of the three axes of the sensor is marked as the (0,0,0) coordinate. The trajectory that the magnet moves is shown in blue describing a closed path around the sensor. Orange circles on the left insets of the figure panel indicate three custom defined target positions of the magnet P1, P2, and P3. The captured trajectory clearly shows that the magnet moved between these defined positions, which demonstrates the capability of the sensor to track the magnet path in 3D. The panel further provides three 2D projections of the 3D trajectory onto the XZ, YZ, and XY planes. These projections better show the movement of the magnet in each plane and also provide a better representation of the sensor capability to capture such movement independently. The orange circles in the 2D projections denote the projected magnet positions at P1, P2, and P3.

### 2.5 Interactive and 4D Mechatronic Applications

**FIGURE 5:**
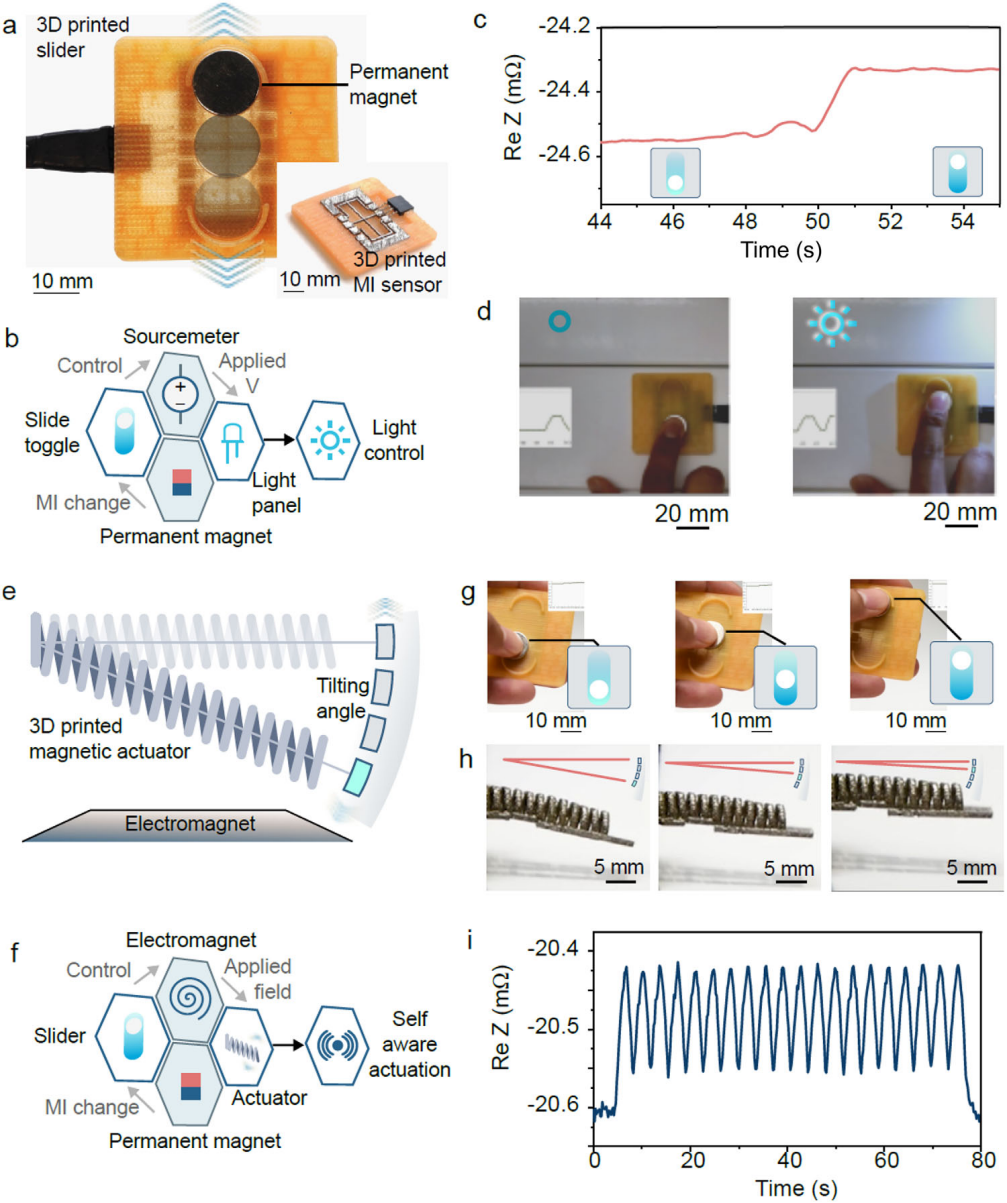
Application scenarios. (a) 3D‐printed toggle switch embedded in a custom polymeric frame for smart home applications. (b) Schematic of LED panel activation using the switch by sliding a permanent magnet along the case, with magnetic attraction to the ${\text{Fe}}_{64}{\text{Ni}}_{36}$ component. (c) Output signal over time generated by the sliding magnet. (d) Demonstration of on/off states of the LED panel. (e) Schematic of a magnetic 4D actuator with a spring‐shaped design. The free tip tilts upon attraction to an electromagnet. (f) Schematic of the actuation principle. (g) Photographs of a user handling a continuous joystick, which controls the inclination of the 4D actuator based on live direct input. (h) Sequence of the tilting of the self‐aware 4D printed mechatronic actuator system with integrated sensing. (i) Time‐resolved signal of the self‐aware actuator during a sinusoidal magnetic field input, illustrating real‐time detection.

In the following, we present several application examples that profited from geometry, magnetic properties, sensing and actuation capabilities of 3D‐printed sensors. Figure 5a introduces a 3D printed toggle switch seamlessly embedded in a custom printed polymeric frame. This structure is activated by a permanent magnet, which is magnetically attracted to the printed sensor and able to slide in order to act as a toggle switch with two distinct states. This binary switch controls the voltage sourced to an illumination panel with a light emitting diode (LED) based on the input received from the sensing part (Figure 5b). The two on/off levels in the output signal (Figure 5c) turn the LED either on or off as shown in Figure 5d. This demonstration illustrates possibilities for custom smart home devices and dedicated control panels (Video S2). The presented remote magnetic interaction may enable the placement of such structures aesthetically and seamlessly integrated behind walls and doors, or other printed parts.

Transitioning to integrated sensing and actuation for 4D mechatronics, an electromechanical magnetically controlled device is introduced in Figure 5e. Mechanical flexibility arises via spring‐like geometry which bends upon applied magnetic fields (maximum applied field is 150 mT). Figure 5f presents the operating principle of this system in a closed loop configuration. The slider with a permanent magnet acts as the controlling element (Figure 5g) modulating the magnetic field of an electromagnet. Its position change, indicated by the magnetoimpedance signal trace, is used as the input to modulate the variation in the magnetic field applied to the spring actuator. This will, in turn, induce a mechanical response of the actuator bending or flexing through the magnetic interaction as shown in Figure 5h. The entire system presented here is a typical form of a closed‐loop operating capability with an actuator able to be remote‐controlled and manipulated via magnetically coupled sliders for finely tuned and dynamic operations (Video S3). Additionally, the actuating structure can be self‐aware of its actuation (Figure 5i), showcasing the output signal of the actuating structure during a periodic motion sequence. Video S4 shows a developed concept of this system integrated as an optomechanical 4D printed part for sweeping a laser, whose beam position is modulated by the interaction of the spring with the magnetic field of an electromagnet. This capability suggests potential applications in optical scanning systems, laser‐based additive manufacturing, and adaptive optics, where integrated sensing and actuation in a single 3D‐printed component reduces complexity both in design and fabrication.

## 3 Conclusions

Here, we report on 3D‐printed magnetoelectronics and demonstrate advantages of integration of magnetic field sensing capabilities into additively manufactured components through laser powder bed fusion of ${\text{Fe}}_{64}{\text{Ni}}_{36}$ alloy. Tailorable structural geometries with unique magnetoimpedance responses and multi‐axis magnetic field detection relying on Hall effect sensing were realized. In both metallurgy and additive manufacturing literature, ${\text{Fe}}_{64}{\text{Ni}}_{36}$ is primarily chosen for its ultra‐low thermal expansion and dimensional stability rather than for sensing [39, 41]. While its soft‐magnetic and magnetostrictive properties are known, there are no reports on 3D‐printed magnetic field sensors based on this alloy. In contrast, high‐sensitivity magnetic sensors typically rely on Permalloy‐type compositions (e.g., ${\text{Fe}}_{20}{\text{Ni}}_{80}$) which are not standard workhorse materials for structural additive manufacturing [45, 47]. Our study demonstrates that 3D‐printed ${\text{Fe}}_{64}{\text{Ni}}_{36}$ can simultaneously meet structural and sensing requirements, delivering high magnetoimpedance response (>300%) that is not typically associated with this alloy nor reported for 3D‐printed sensor architectures. This convergence is crucial for “structural electronics”, as the same alloy used for its mechanical properties now also enables viable magnetic field sensing, avoiding reliance on non‐structural, non‐AM‐friendly compositions. Given that 3D processing greatly simplifies the integration with structural parts and electronics operating at lower frequencies than those compared to traditional giant magnetoimpedance sensors [34, 45, 46, 47], our approach improves scalability and integration degree of sensing in constructed parts. The magnetic actuation of spring‐shaped components introduces representative examples of magnetically responsive 4D‐printed parts with sensing and actuation, offering new possibilities in interactive devices, touchless interfaces, and 4D mechatronic systems.

Compared to approaches based on fabricating sensors on flexible substrates using conventional thin‐film deposition, or mounting miniaturized rigid sensors onto flexible carriers, our 3D‐printed approach enables structural electronics: the sensor is within the structure itself, so there are no assembly weak points and mechanical robustness is inherently higher than for glued or laminated sensors. At the same time, 3D printing lets us realize truly volumetric, complex geometries (e.g., 3D Hall‐type or magnetoimpedance architectures with out‐of‐plane current paths) that are fundamentally inaccessible to planar thin‐film processes or to simply mounting miniaturized rigid sensors on a flexible carrier. Notably, the sensing elements reported here are bulk 3D‐printed structures rather than single‐layer ferromagnetic films. While magnetoimpedance in thin films and wires can be enhanced by multilayer or sandwich architectures (e.g., FM/conductor/FM), translating such stacks to metallic additive manufacturing is currently challenging because multimaterial 3D printing is still an emerging technology. In the future, advances in multimaterial metal 3D printing could enable composite architectures (e.g., a highly conductive core with a soft‐magnetic FeNi cladding), which may further enhance the sensor performance.

This work is expected to stimulate further explorations of different 3D printing techniques for the fabrication of 3D‐printed magnetic field sensors of complex sensor geometries aiming on enhanced multi‐axis sensing selectivity. We expect that future efforts will focus in multimaterial printing towards multifunctionality and tailored electromechanical properties combined with 4D‐printing technologies, magnetically assisted assembly, and compliant mechanisms [52]. Further improvements in the sensor performance are envisioned to come with advances in new families of 3D‐printable magnetosensitive materials and involving machine learning methods for sensor calibration [53, 54]. The possibility to concurrently fabricate geometric shape, functionality, and actuation capabilities makes 3D‐printed magnetosensitive devices a versatile platform that opens up appealing perspectives like systems able to measure energy consumption in 3D‐printed houses and 3D‐printed compartments in prospective space missions [55, 56]. Furthermore, this work opens opportunities for the development of distributed sensing networks for structural health monitoring, biocompatible implants and prosthetics with embedded activity tracking [57, 58, 59]. In this respect, 4D‐printing combined with magnetic field sensing will further enable out‐of‐the‐printer electromechanical parts for robots, human‐machine interfaces, prosthetics, multi‐axis motors, where custom reformable structural objects are responsive entities inherently aware of their state and their surroundings.

## 4 Experimental Section

### 4.1 Feedstock Powder and Laser Powder Bed Fusion Process

Gas atomized ${\text{Fe}}_{64}{\text{Ni}}_{36}$ powders are available through the Thyssenkrupp Materials Trading GmbH (Essen, Germany). The morphology and chemical composition of the powder were analyzed with a scanning electron microscope (SEM‐JSM 6610 LV) at 15 kV equipped with an energy‐dispersive x‐ray spectrometer (EDS, Oxford instruments X‐Max 80). The particle size distribution was measured with a Retsch Technology CAMSIZER X2 (ISO 13322‐2). The rheology of the powder was measured by means of flow rate and the shear properties with a powder‐rheometer from Freemantech (Tewkesbury, UK). X‐ray diffraction (XRD) analyses were carried out using a Rigaku SmartLab 3 kW in the Bragg–Brentano geometry with a Cu anode, a Ni filter and a HyPix‐3000 detector in XRF reduction mode.

A laser powder bed fusion system (Renishaw AM400, Stone, UK) was used for the fabrication of 3D‐printed parts. The system is equipped with an Yb:YAG laser and has a build volume of 245×245×285 ${\mathrm{mm}}^{3}$. The laser has a maximum power of 400 W and a beam diameter of 70 μm. All printing trials were conducted under argon atmosphere to minimize oxidation.

The printing optimization started with the deposition of single tracks of 8 mm length. A full factorial design (4×3×3) was carried out with laser power (250/300/350/400 W), point distance (40/60/80 μm), and exposure time (60/80/100 μs) as main parameters. Two single tracks were printed for each set of conditions to observe track continuity, energy density effects, and potential defects. The goal of this stage was to determine suitable exposure time and point distance ranges that would yield continuous, defect‐free tracks, and to identify laser powers causing insufficient melting or excessive energy input (Table S1). Based on these results, we fixed the point distance and exposure time at 40 μm and 80 μs, respectively, and performed a central composite circumscribed (CCC) design (3×3) focusing on laser power in the range 300–400 W and hatch distance in the range 110–150 μm. These fabrication trials aimed to optimize fabrication conditions for printing cube‐like objects while monitoring porosity fluctuations and melt pool stability. The results indicated optimal laser power and hatch distance combinations for producing near‐full density parts without introducing keyholes or cracks. Based on these results, we fixed the laser power at 375 W and a 3×3 factorial design was implemented by varying the exposure time (80/90/100 μs) and hatch distance (90/110/130 μm). Printed cubes produced under these conditions were evaluated for relative density, defect formation, and cross‐sectional homogeneity. The rationale of this stage was to refine the process window, select the parameter set yielding the highest density, and validate reproducibility of the printing process. The final optimized parameters enabling prints of cube‐like objects at relative density of 99.95% are as follows: exposure time: 100 μs, hatch distance: 110 μm, and volumetric energy density: 94.7 J ${\mathrm{mm}}^{-3}$.

### 4.2 Morphological Characterization

The top surface of line tracks was analyzed by a Keyence VHX5000 microscope. The density of 3D‐printed cubes was measured on the cross‐section areas (parallel and perpendicular to the building direction) by an Olympus GX51 optical microscope. The relative density/porosity was measured by Olympus Stream Enterprise software. The microstructure of the samples printed at optimal conditions was investigated by SEM, and the surface roughness was studied by a confocal microscope Keyence VK‐X200.

### 4.3 Mechanical Properties

Vickers hardness measurements were performed on the cross‐section areas of the printed cubes with an AMH‐43 microhardness tester (LECO Instrumente GmbH, Mönchengladbach, Germany) according to DIN EN ISO 6507. Tensile tests were performed according to DIN 50125. For that, cylindrical parts with diameter 9 mm and length 43 mm were printed at different orientations (Figure S7). A minimum of 7 samples per print orientation were manufactured and tested. Later on, structural supports were removed and samples were machined according to DIN 50125 for tensile testing.

### 4.4 Magnetic Characterization

#### 4.4.1 Magnetoimpedance Measurements

Magnetoimpedance measurements were performed using an OMICRON Lab Bode 100 vector network analyzer configured in both Voltage mode and Shunt (impedance adapter) mode for very low‐impedance devices under test. The frequency range was swept from 1 kHz to 1 MHz with an AC excitation voltage of 100 mV. Magnetic fields were applied in the range of ±500 mT using an electromagnet. The magnetic field was applied in the plane along the long axis of the sensor. Field steps were synchronized to frequency sweeps using a LabVIEW‐controlled software.

3D‐printed sensors of various geometries were contacted via spring‐loaded conductive tips or direct welding for permanent electrical contacts. All measurements were performed at ambient laboratory temperature. The impedance modulus |Z(H,f)| was baseline‐subtracted and normalized to its value at H=500 mT for data analysis. The normalized magnetoimpedance ratio was calculated as:

$$Z_{\mathrm{norm}}\left(H,f\right)=\frac{\left|Z(H,f)|-|Z(500\;\text{mT},f)\right|}{\left|Z(500\;\text{mT},f)\right|}$$

We also checked the DC magnetoresistance of the 3D‐printed sensors. However, due to their extremely low resistance, the DC magnetoresistance signal was close to the noise floor and hardly measurable. This is consistent with the negligible magnetoimpedance response observed at low frequencies (see Figure 1e, 1 kHz).

#### 4.4.2 Hall Effect Measurements

The Hall effect characterization was performed using a Tensormeter device (HZDR Innovation GmbH, Germany) operating in the zero offset Hall mode, with a switching bias current of about 20 mA. The measurement system was integrated with a USB DAQ 6211 (National Instruments) and LabVIEW for automated control and data acquisition. The 3D‐printed parts were fabricated with a Hall bar structure and dedicated contacts for the measurement.

During measurements, both DC (±2 V) and AC (775 Hz) excitations were applied with the Hall voltage detected by the Tensormeter device. An external magnetic field was swept from −50 to +50 mT at room temperature. The vector component analysis was achieved using the zero offset Hall technique with a switching current scheme, which efficiently suppresses offset voltages and enhances the measurement stability.

#### 4.4.3 Integral Magnetometry Measurements

Magnetic hysteresis loops were measured using a Lake Shore 7407 vibrating sample magnetometer (VSM) in the field range of ±0.2 T with a field sweep rate of 5‐10 mT $s^{-1}$. The measurement was done at 300 K.

### 4.5 Demonstrators

#### 4.5.1 Three‐Axis Magnetic Field Tracking

A 3D‐printed 3D Hall cross was fabricated with current paths routed in three orthogonal planes and six soldered contacts (two per axis) for electrical interfacing. For mechanical protection and ease of handling, the device was embedded in a Sylgard polydimethylsiloxane (PDMS) cube, which was cast in a polymeric container and cured at room temperature for 24 h. During experiments, the north pole of a 10 mm button‐shaped permanent magnet was moved outside the PDMS packaging, traveling among defined positions (P1–P2‐P3) outside the cube boundaries. The three axes (X,Y,Z) were aligned with the orthogonal current paths, with the geometric center of the Hall cross being at the origin. Further details of the experiment are shown in Video S1.

The Hall cross was operated in the switching‐current, zero‐offset mode using a Tensormeter device connected to the six sensor leads of the 3D‐printed 3D Hall effect sensor. AC excitation at 775 Hz (optimized for minimal offset and noise) was combined with a DC bias voltage tuning for the offset minimization. The changes of the Hall resistance, $\Delta R_{H,i}$, were acquired for each sensor axis i∈{x,y,z}. The calibration was performed by mapping the Hall resistance span (±10μΩ) to the half‐span distance from the origin to each of the PDMS cube faces (w/2, l/2, h/2), yielding real‐time 3D coordinates according to the equation:

$$i\left(\mathrm{mm}\right)=-\frac{\Delta R_{H,i}}{R_{\mathrm{span},\max}}\times \frac{D_{i}}{2},\;R_{\mathrm{span},\max}=\pm 10\;\mu \Omega ,$$

where $D_{i}$ is the device dimension along each axis. The coordinates beyond $\pm D_{i}/2$ indicate that the magnet is outside the PDMS volume. The data were sampled at 50 ms intervals, with five consecutive samples averaged per point, and visualized in real time using a custom LabVIEW‐based user interface. The 3D‐printed sensor reconstructed the 3D trajectory of a magnet and its projections onto the principal planes. A cross‐talk between axes due to field focusing effects was minimized through frequency and offset optimization. Embedding the device in PDMS provided mechanical robustness and facilitated touchless tracking, highlighting future potential for gesture recognition, robotics, and biomedical applications.

#### 4.5.2 Joystick and Switching Demonstrators

For joystick and switching demonstrations, a 0.5 mm thick Hall cross structure was integrated into a fully additively manufactured slider switch. The housing was printed using a PLA printer, featuring a 10 mm diameter sliding channel accommodating a button‐shaped NdFeB permanent magnet. Magnetostatic interactions between the permanent magnet and the 3D‐printed magnetic bar enabled a fixture‐free sliding mechanism, illustrating the potential of combining shape, sensing, and magnetic actuation in 3D‐printed human–machine interfaces. The normalized magnetoimpedance amplitude at 20 kHz,

$$A_{\mathrm{norm}}\left(t\right)\in \left[0,1\right],$$

was used both to control an LED light via

$$\begin{array}{cc} V_{\mathrm{LED}}\left(t\right) & =V_{\min}+\left(V_{\max}-V_{\min}\right),\\ A_{\mathrm{norm}}\left(t\right) & =1\;V+8\;V\times A_{\mathrm{norm}}\left(t\right), \end{array}$$

and to drive the electromagnet for 4D actuation. The signal acquisition and normalization were performed via a custom LabVIEW program using a USB DAQ 6211 (National Instruments). Further details of the experiment are summarized in Video S2.

#### 4.5.3 4D Actuation Demonstration

For 4D actuation experiments, the normalized signal $A_{\mathrm{norm}}\left(t\right)$ was fed to a power supply of an electromagnet (ITS‐MS‐7040‐12VDC, Intertec) providing magnetic field of up to 50 mT. Field ramps from 0 to 40 mT were generated according to

$$B\left(t\right)=B_{\max}\times A_{\mathrm{norm}}\left(t\right)=40\;\text{mT}\times A_{\mathrm{norm}}\left(t\right).$$

The 3D‐printed actuator shaped as a helical spring underwent field‐induced deformation compatible with 4D‐printing concepts. The actuator was positioned between poleshoes of an electromagnet with an integrated LED module projecting onto a custom screen. The magnetic field was modulated using a sinusoidal sequence controlled by a LabVIEW software, and displacement was monitored optically with a video camera.

## Author Contributions

ESO‐M, CL, LS, and DM conceptualized the study. ESO‐M and AMV carried out the formal analysis. LS, CL, and DM secured funding. Investigation was performed by ESO‐M, AMV, and FG, and methodology was developed by ESO‐M, AMV, FG, and DM. Concepts for the demonstrators were developed by ESO‐M and DM with the support of CL and LS. Project administration and supervision were provided by LS, CL, and DM. Software implementation was done by ESO‐M, and visualizations were prepared by ESO‐M and AMV. ESO‐M, AMV and DM wrote the original draft of the manuscript, and all authors contributed to reviewing and editing the final version.

## Conflicts of Interest

The authors declare no conflicts of interest.

## Supporting information


**Supporting File 1**: advs75985‐sup‐0001‐SuppMat.pdf.

**Supporting File 2**: advs75985‐sup‐0002‐VideoS1.mp4.

**Supporting File 3**: advs75985‐sup‐0003‐VideoS2.mp4.

**Supporting File 4**: advs75985‐sup‐0004‐VideoS3.mp4.

**Supporting File 5**: advs75985‐sup‐0005‐VideoS4.mp4.

## Data Availability

All of the data supporting the conclusions are available within the article and the Supplementary Information. Additional data are available from the corresponding authors upon reasonable request.
