# Supplementary material for: 3D‐Printed Magnetoelectronics for Interactive Appliances and Self‐Aware 4D‐Printed Mechatronics
Source: Adv Sci (Weinh). 2026 Jun 13;13(49):e75985. doi: 10.1002/advs.75985 (PMC13336971; doi:10.1002/advs.75985)
Supplement: Supplementary file 1 [file ADVS-13-e75985-s001.pdf]

Supporting Information for

3D-printed magnetoelectronics for interactive electronics and self-aware 4D-printed mechatronics

Eduardo Sergio Oliveros-Mata,[1] Anna Martin Vilardell,[2] Fabian Ganss,[1] Christoph Leyens,[2,3] Lukas Stepien,[2] and Denys Makarov[1,*]

[1] *Helmholtz-Zentrum Dresden-Rossendorf e.V., Institute of Ion Beam Physics and Materials Research, 01328 Dresden, Germany*
[2] *Fraunhofer Institute for Material and Beam Technology IWS, 01277 Dresden, Germany*
[3] *Technische Universität Dresden, Institute of Materials Science, 01069 Dresden, Germany*

CONTENTS

Supporting Videos 2

Supporting Table 3

Supporting Figures 4

* d.makarov@hzdr.de

SUPPORTING VIDEOS

Supporting Video 1. **Three-Axis Magnetic Field Tracking.** This video demonstrates real-time three-dimensional magnetic field tracking using a monolithic 3D-printed 3D Hall cross sensor. The setup showcases the sensor ability to independently detect all three orthogonal components of an external magnetic field vector. During the demonstration, a permanent magnet is moved through space and the 3D-printed sensor reconstructs its trajectory in real time, visualizing the 3D path. The video highlights the potential for precise vector field mapping in advanced human-machine interfaces.

Supporting Video 2. **Magnetic Slider Switch Demonstration.** This demonstration features a 3D-printed Hall cross integrated into a slider switch, functioning as a magnetic toggle. The video illustrates actuation and magnetic field sensing: a permanent magnet is used to activate the switch, which in turn controls a panel with light emitting diode (LED).

Supporting Video 3. **Joystick and 4D Actuator Demonstrator.** This video presents a continuous joystick interface fabricated via 3D printing, where user input modulates the inclination of a spring-shaped 4D actuator. The demonstration underscores the synergy between 4D printing, actuation, and integrated sensing for advanced 4D mechatronics.

Supporting Video 4. **Optomechanical 4D-Printed Actuator Demonstration.** A demonstration of an optomechanical 4D-printed actuator, showing magnetic field-induced deformation and optical displacement tracking. The video captures the actuator tilting under magnetic stimulus and the simultaneous optical monitoring of its displacement when a laser beam sweeps a white screen under a sinusoidal motion pattern.

SUPPORTING TABLE

Supporting Table I. Summary of the printing parameters and respective SEM micrographs showing line-shaped printed structures upon adjustment of the L-PBF printing process.

Laser power [W]	250					300	350	400
Exposure time [µs]	60			80	100			
Point distance [µm]	40	60	80	40				
Line energy density [J/m]	0.375	0.25	0.1875	0.5	0.625	0.75	0.875	1

Tests

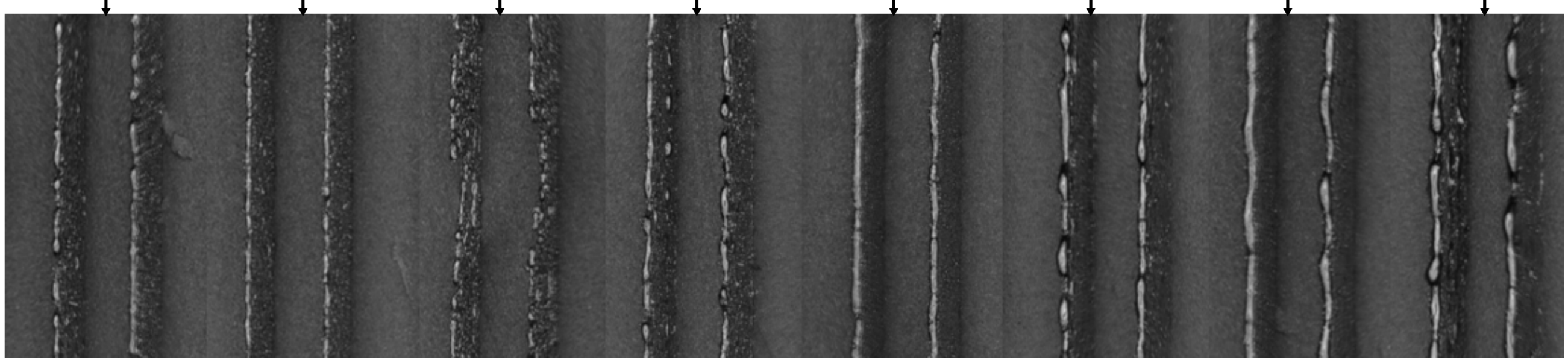

SUPPORTING FIGURES

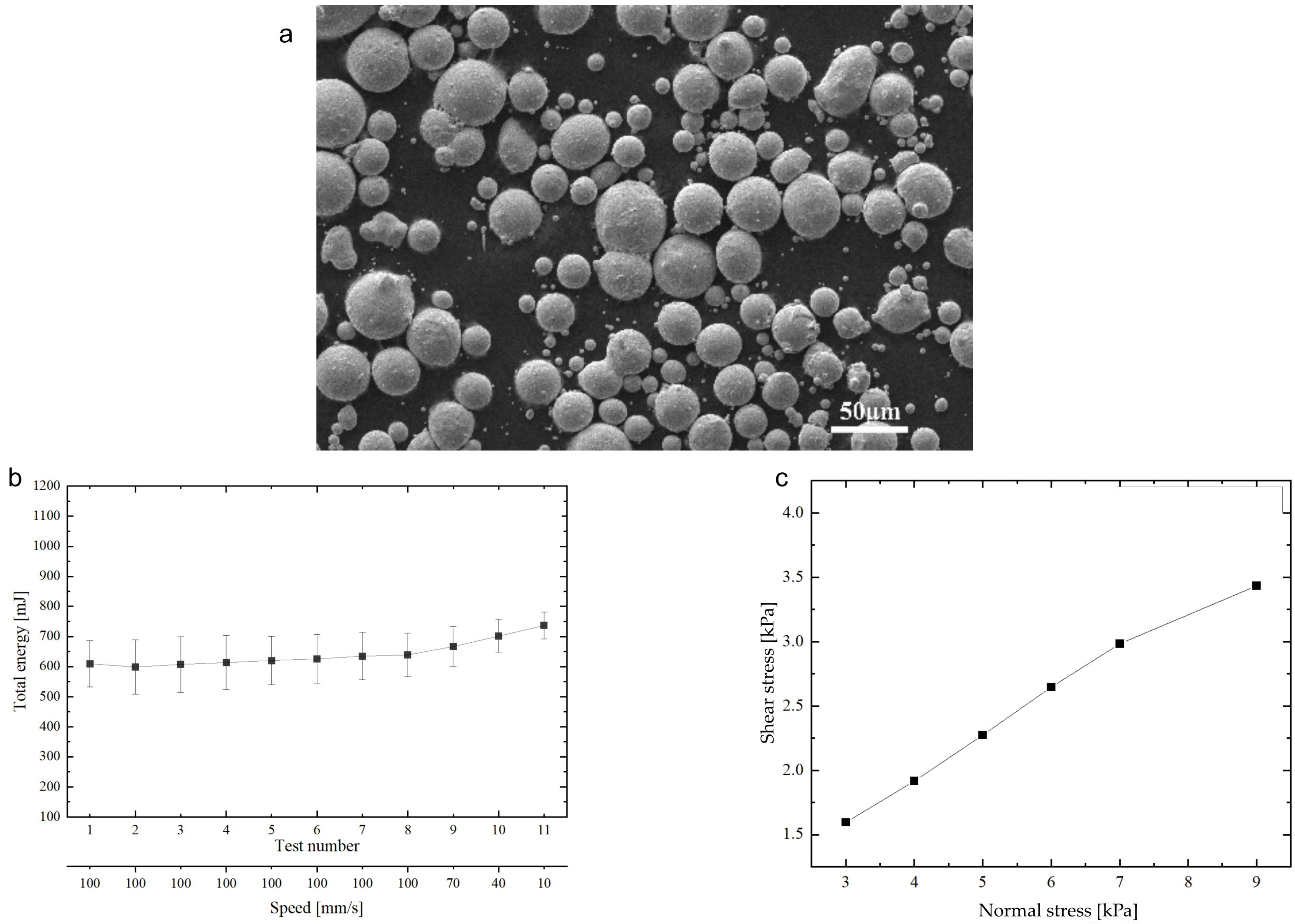

Supporting Fig. 1. **Characterization of the $Fe_{64}Ni_{36}$ alloy powder.** (a) SEM micrograph showing spherical powder morphology with occasional satellites ($d_{10} = 29.4\,\mu\text{m}$, $d_{50} = 52.7\,\mu\text{m}$, $d_{90} = 95.5\,\mu\text{m}$). (b) Flow-energy curve from the powder rheometer. The data reveals stable flow energy throughout agitation. (c) Shear-cell test: cohesive index vs. normal stress indicating low inter-particle cohesion (no significant attachment).

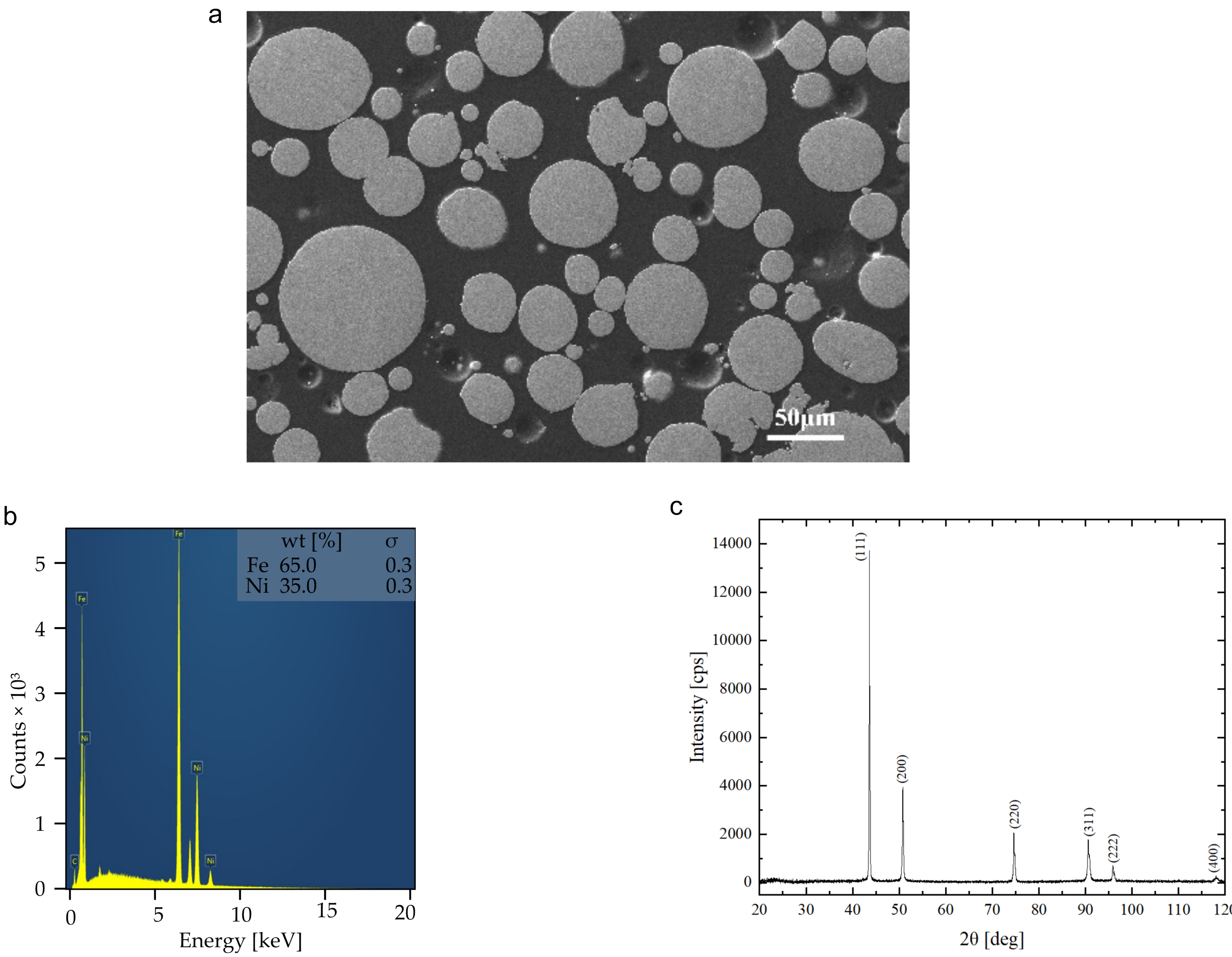

Supporting Fig. 2. **Cross-section characterization of the $Fe_{64}Ni_{36}$ powder.** (a) Back-scattered SEM image of a frozen section through an assembly of individual $Fe_{64}Ni_{36}$ alloy particles revealing their dense, core-solid morphology. (b) EDS analysis on the cross-section shown in panel (a) revealing the alloy composition of 65 wt.% Fe and 35 wt.% Ni. The measured value is slightly Fe-rich but within expected EDS uncertainty without an internal standard. (c) XRD pattern of the $Fe_{64}Ni_{36}$ powder, matching the fcc γ-phase of the material (ICDD PDF card 00-047-1405), thus confirming the expected composition with no detectable bcc α-phase reflections.

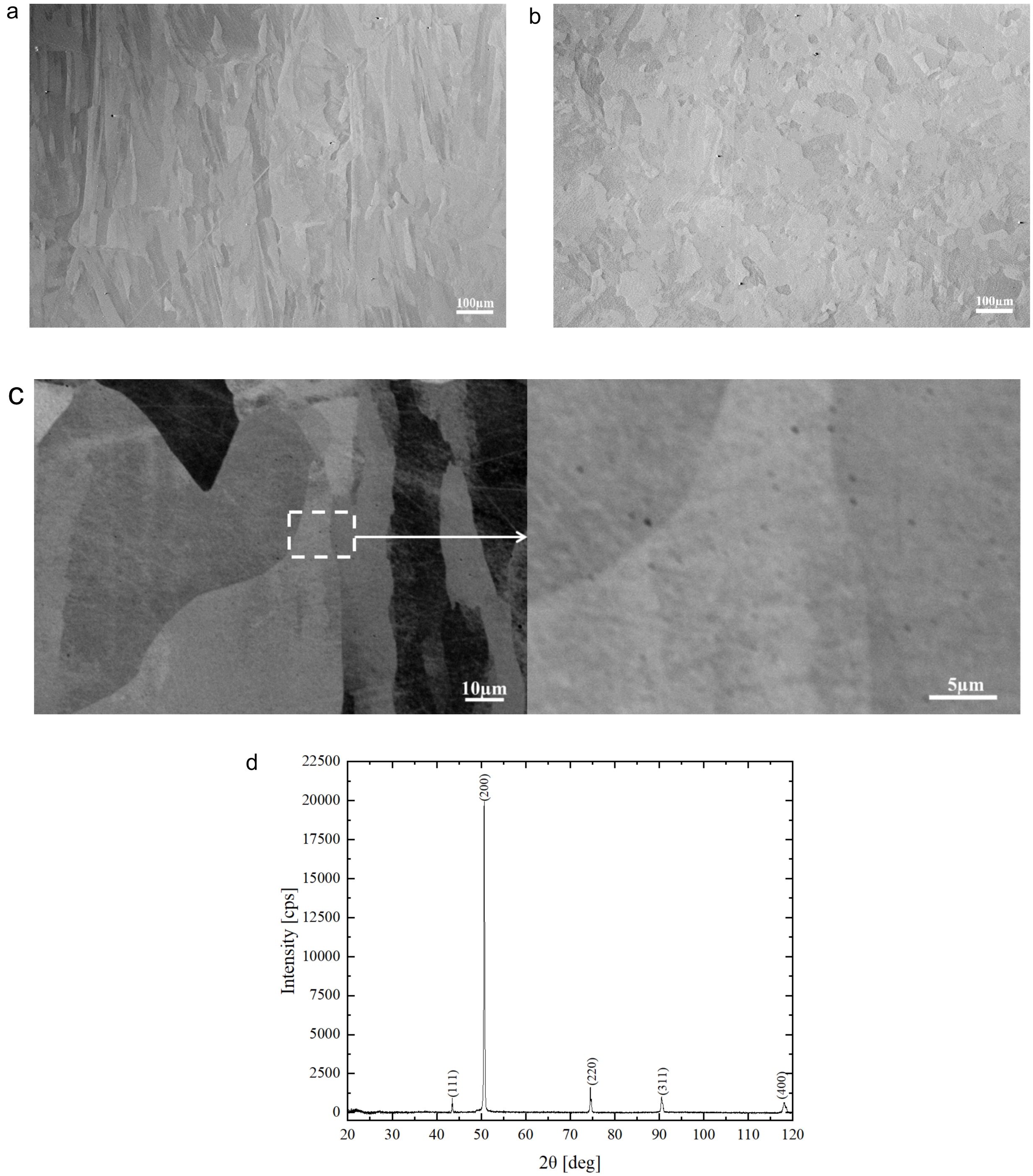

Supporting Fig. 3. **Microstructural characterization of 3D-printed $Fe_{64}Ni_{36}$ cubes.** (a) Low-magnification back-scattered SEM micrograph of a cube cross-section with the cut parallel to the build direction, showing columnar grains ($<$ 100 µm) grown along the thermal gradient. (b) Low-magnification back-scattered SEM micrograph of a cross-section with the cut perpendicular to the build direction. (c) High-magnification back-scattered SEM micrographs showing sparse nanometric precipitates that might correspond to the bcc α-phase and are below the XRD detection limit. (d) Corresponding XRD pattern revealing a pronounced (100) texture, suppressed (111) orientation and indicating the retained fcc γ-phase of the $Fe_{64}Ni_{36}$ alloy.

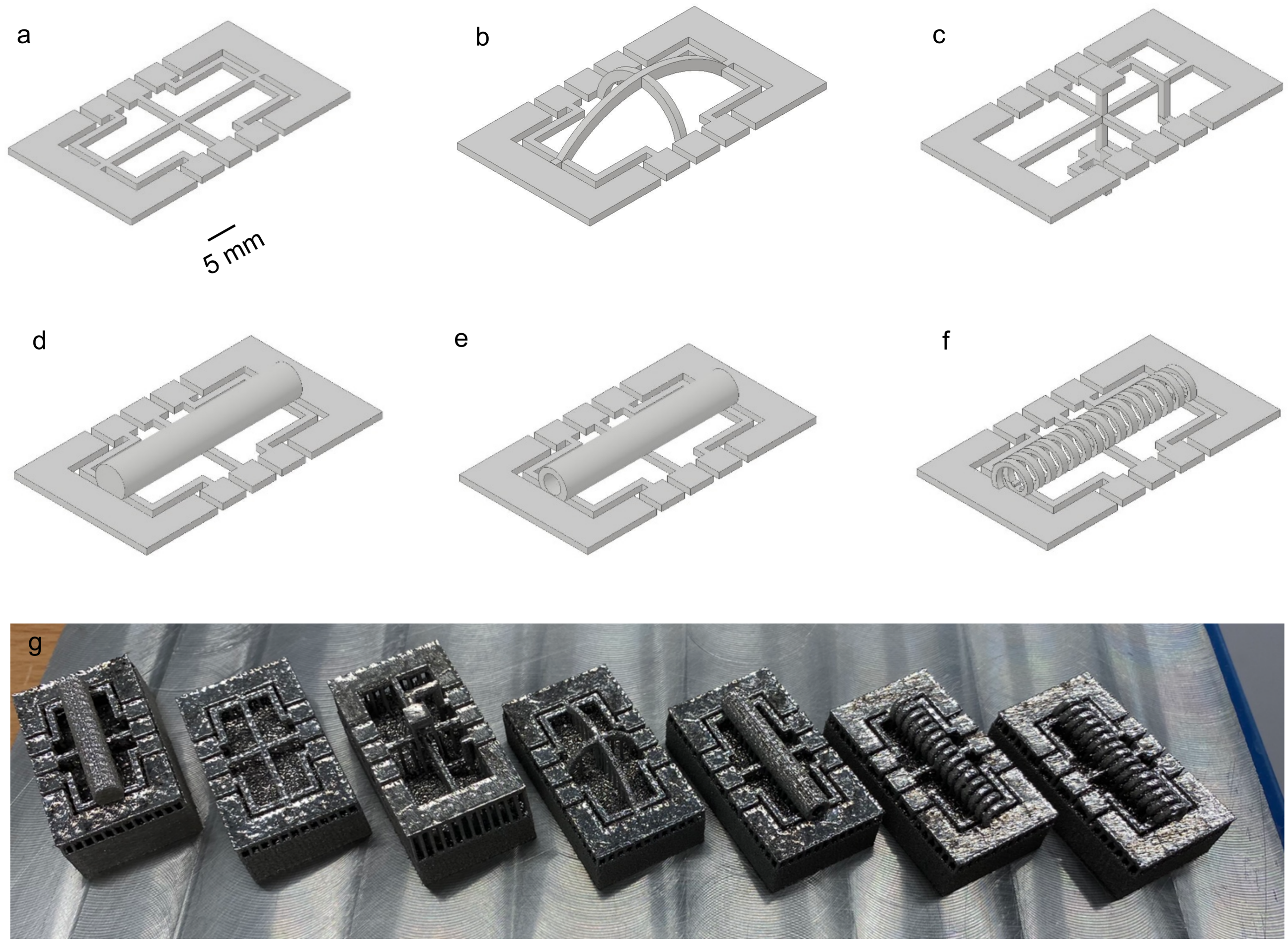

Supporting Fig. 4. **Designs of 3D-printed magnetic field sensors.** (a) Hall cross shape. (b) Curved Hall cross. (c) Three-dimensional Hall cross. (d) Solid cylinder with diameter of 5 mm. (e) Hollow tube with outer diameter of 5 mm and inner diameter of 3 mm. (f) Helical coil with 15 turns; outer diameter: 5 mm, inner diameter: 3 mm. The shown geometries were printed with $Fe_{64}Ni_{36}$ at 0.5 mm and 1 mm thickness to assess thickness effects on magnetic response. (g) Optical micrographs of several fabricated samples of different geometries.

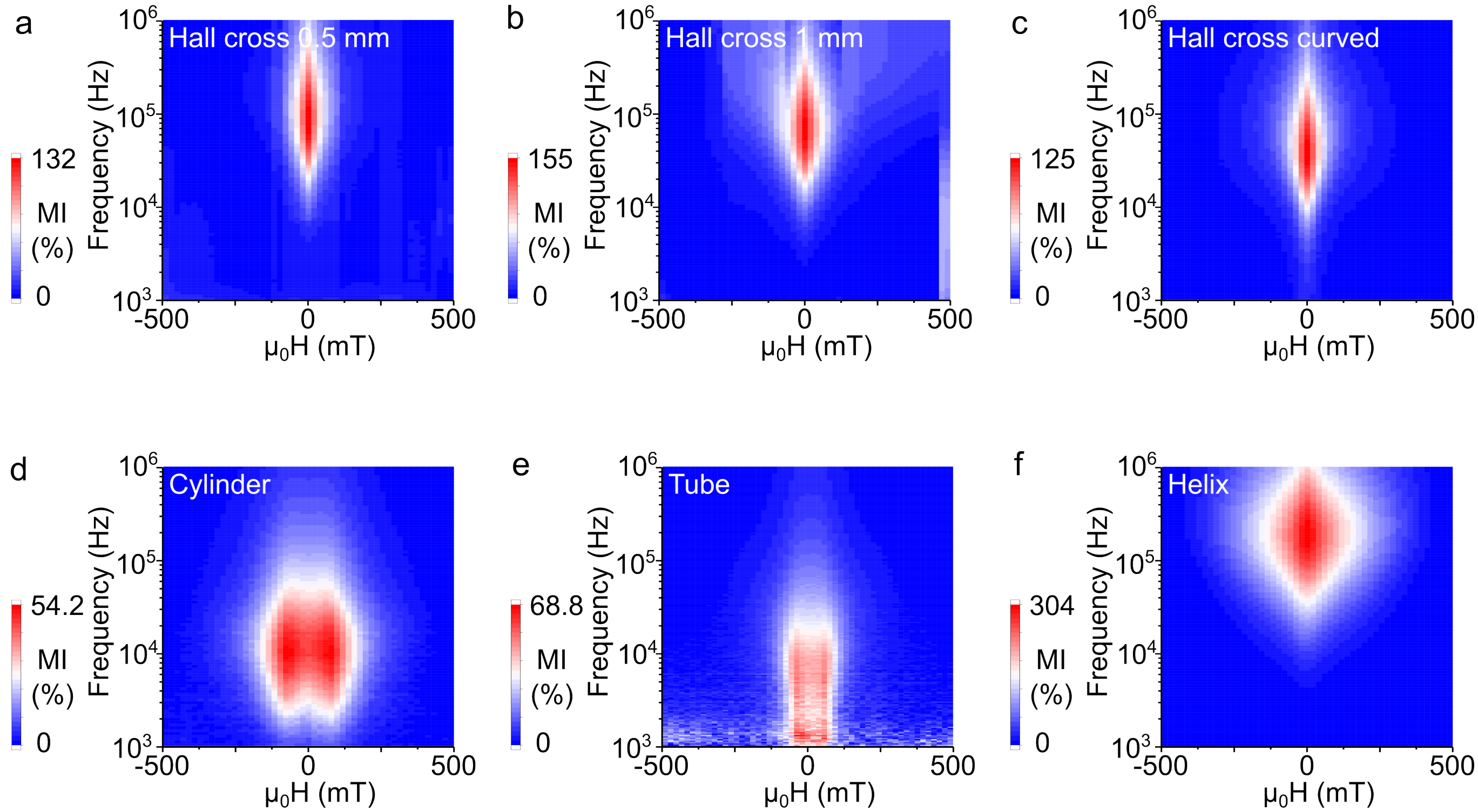

Supporting Fig. 5. **Magnetoimpedance performance of 3D-printed sensors.** The measurement is done in a 4-point configuration using two outer pairs of contacts prepared for each sensor geometry shown in Supporting Figure 4. (a) Straight sensor stripe of 0.5 mm thick (geometry shown in Supporting Figure 4a). (b) Straight sensor stripe of 1.0 mm thick (geometry shown in Supporting Figure 4a). (c) Curved sensor stripe of 0.5 mm thick (geometry shown in Supporting Figure 4b). (d) Straight sensor cylinder with diameter of 5 mm (geometry shown in Supporting Figure 4d). (e) Straight sensor tube with outer diameter of 5 mm and inner diameter of 3 mm (geometry shown in Supporting Figure 4e). (f) Sensor shaped as a helical coil with 15 turns; outer diameter: 5 mm, inner diameter: 3 mm (geometry shown in Supporting Figure 4f). The thickness of the printed structure is 0.5 mm.

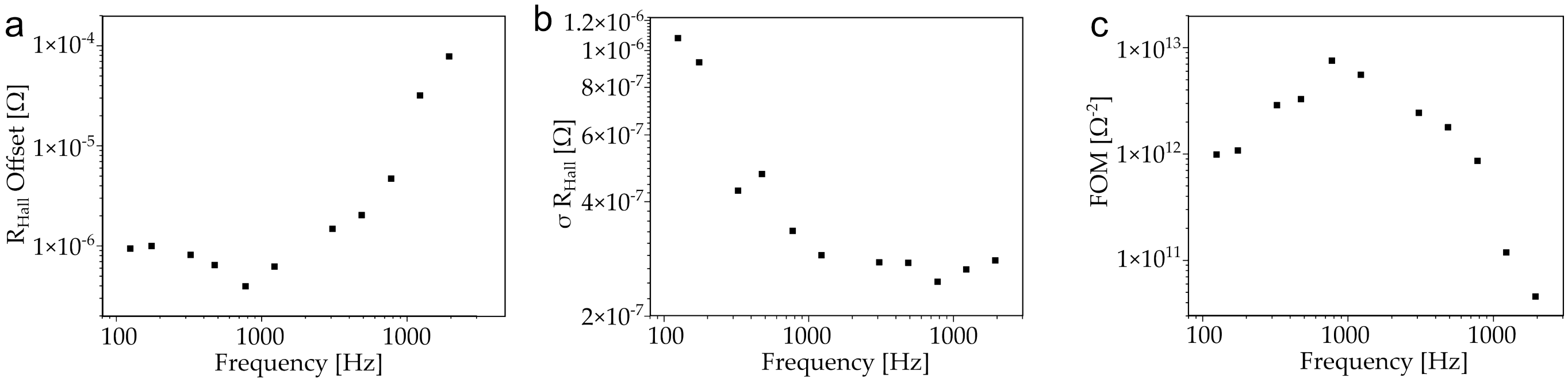

Supporting Fig. 6. **Frequency optimization for Hall effect measurements.** (a) Offset of the Hall resistance at different driving frequencies. (b) Standard deviation of the Hall resistance signal over 30 s acquisition time at different driving frequencies. (c) Figure of merit (FOM) defined as the inverse of the product of the offset of the Hall resistance and standard deviation of the Hall resistance signal over 30 s acquisition time. Larger values of the FOM metric indicate better performance (minimized offset and noise deviations).

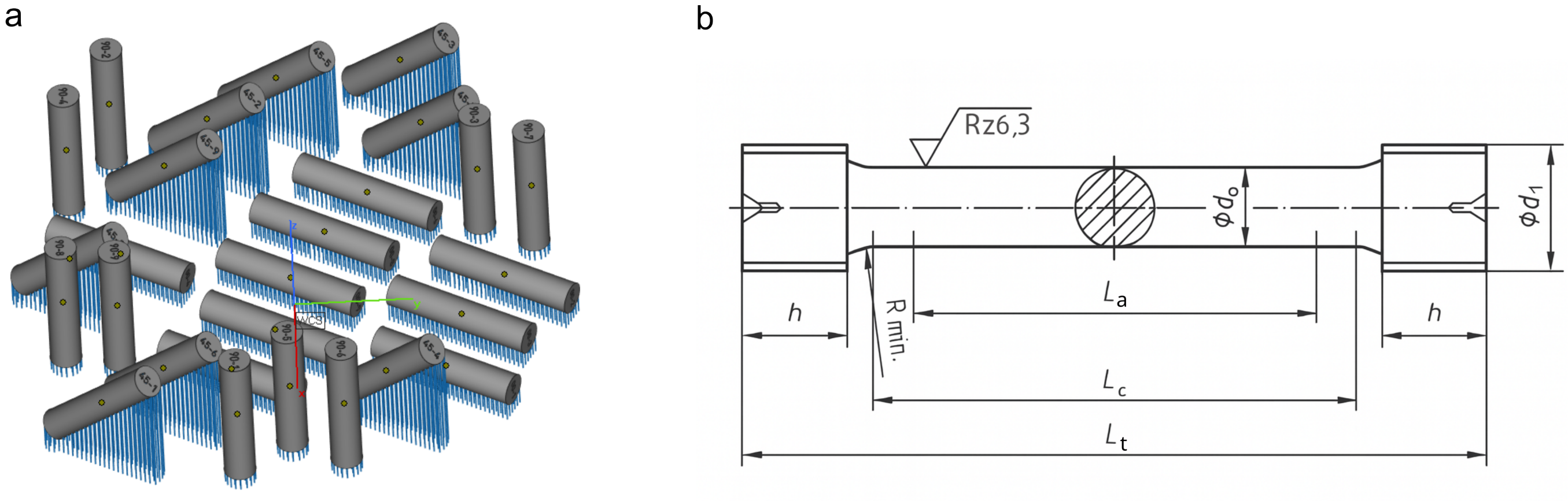

Supporting Fig. 7. **Geometry of L-PBF 3D-printed test specimens.** (a) 3D model of the printed test specimens, which were fabricated at different orientations (XY, ZY, as well as 45°). A minimum of 7 samples per orientation were manufactured and tested. Structural supports (indicated with blue lines) were later removed and samples were machined according to DIN 50125 for tensile testing. (b) Computer aided design (CAD) drawing of the specimen geometry used for the tensile testing. The structure is prepared according to the DIN 50125.
